# Surface Treatments and Adhesives Used to Increase the Bond Strength Between Polyetheretherketone and Resin-based Dental Materials: A Scoping Review

**DOI:** 10.3290/j.jad.b2288283

**Published:** 2022-05-16

**Authors:** Pablo Soares Machado, Ana Carolina Cadore Rodrigues, Eduardo Trota Chaves, Alexandre Henrique Susin, Luiz Felipe Valandro, Gabriel Kalil Rocha Pereira, Marília Pivetta Rippe

**Affiliations:** a PhD Student, MSciD and PhD Postgraduate Program in Oral Science, Faculty of Dentistry, Federal University of Santa Maria (UFSM), Santa Maria, Rio Grande do Sul State, Brazil. Study conception and design, search, screening and charting of the data, analysis, article writing and review.; b PhD Student, MSciD and PhD Postgraduate Program in Oral Science, Faculty of Dentistry, Federal University of Santa Maria (UFSM), Santa Maria, Rio Grande do Sul State, Brazil. Study design, search, screening and charting of the data, review.; c PhD Student, Post-Graduate Program in Dentistry (PPGO), Federal University of Pelotas – UFPel, Pelotas, Rio Grande do Sul State, Brazil. Study design, search, screening and charting of the data, review.; d Full Professor, MSciD and PhD Postgraduate Program in Oral Science, Faculty of Dentistry, Federal University of Santa Maria (UFSM), Santa Maria, Rio Grande do Sul State, Brazil. Study conception and design, review.; e Associate Professor, MSciD and PhD Postgraduate Program in Oral Science, Faculty of Dentistry, Federal University of Santa Maria (UFSM), Santa Maria, Rio Grande do Sul State, Brazil. Study conception and design, interpretation of data, review.; f Adjunct Professor, MSciD and PhD Postgraduate Program in Oral Science, Faculty of Dentistry, Federal University of Santa Maria (UFSM), Santa Maria, Rio Grande do Sul State, Brazil. Study conception and design, interpretation of data, review and final approval.; g Adjunct Professor, MSciD and PhD Postgraduate Program in Oral Science, Faculty of Dentistry, Federal University of Santa Maria (UFSM), Santa Maria, Rio Grande do Sul State, Brazil. Study conception and design, interpretation of data, review.

**Keywords:** adhesion, PEEK, surface modification, resin cement, resin composite

## Abstract

**Purpose:**

To identify and discuss the available surface treatments and adhesives for polyetheretherketone (PEEK) to increase its bond strength to resin-based materials used in dentistry.

**Materials and Methods:**

The reporting of this scoping review was based on PRISMA. The study protocol was made available at: https://osf.io/4nur9/. Studies which evaluated PEEK surface treatments and its bond strength to resin-based materials were selected. The search was performed in PubMed, Scopus, Web of Sciences and Cochrane databases. The screening was undertaken by 3 independent researchers using the Rayyan program. A descriptive analysis was performed considering study characteristics and main findings (title, data of publication, authors, PEEK characteristics, surface treatments, control group, bonded set, luting agent, specimen geometry, storage, thermocycling, pre-test failures, test geometry, failure analysis, main findings, and compliance with normative guidelines).

**Results:**

The initial search yielded 1965 articles, of which 32 were included for descriptive analysis. The review showed that the use of surface treatments and adhesives are important to promote bond strength to PEEK. Up until now, various surface treatments have been explored for bond improvement to PEEK. Sulfuric acid etching is commonly reported as promoting the highest bond strength, followed by alumina-particle air abrasion. Regarding adhesives, the use of a specific adhesive containing MMA, PETIA (pentaerythritol triacrylate), and dimethacrylates yields the best adhesive performance.

**Conclusion:**

Sulfuric acid etching and alumina-particle air abrasion followed by application of bonding agents containing MMA, PETIA and dimethacrylates are the most effective choices to increase resin-based materials’ adhesion to PEEK.

Prosthetic reconstructions using implant-supported crowns are becoming very common in dental practice. In this sense, even though the use of titanium abutments is already established as successful, the high esthetic demand of patients has encouraged the use of metal-free abutments to avoid the grayish aspect of thin marginal tissues around the metal.^18^ Therefore, the use of polyetheretherketone (PEEK) has been explored among the existing options. PEEK is a high performance, thermoplastic, polymeric material,^24^ which has been considered promising in dentistry based on its high biocompatibility,^21^ ease of milling (reduced processing time), and low cost in comparison to other metal-free options (ie, dental ceramics). In fact, a recent study showed that PEEK may be a suitable alternative to zirconia abutments considering their mechanical behavior in fatigue testing.^3^

In addition to the importance of the material itself, the need for studies evaluating luting agents and adhesion ability of dental materials is also clear,^5^ since the bonding interface integrity is also important for the mechanical behavior of a restorative set-up in attempting to reduce the risk of premature failures.^39^ For luted implant-supported crowns, the restoration must be luted with a resin cement over the PEEK abutment. In addition, PEEK must be veneered with composite resin when used as a substructure for a milled crown due to its opaque color in order to be luted to the tooth or implant abutment. Nevertheless, increased bonding between PEEK and resin-based materials is still considered challenging, because PEEK presents a complex chemical nature and poor wettability, resulting in low surface energy and resistance to surface changes.^19^ Thus, approaches to optimize the adhesive ability of this system must be explored, and some studies have evaluated the effect of different surface treatment methods on surface topographical changes (relevant for micromechanical bonding), the use of bonding agents (relevant for interlocking and chemical bonding), and/or luting agents to improve the resin bond to PEEK.^6,30,36,40,41,42^

According to Silthampitag et al,^28^ the major factor in promoting better adhesion between PEEK and resin-based materials appears to be inducing micromechanical interlocking between such substrates. From this viewpoint, they showed that sulfuric acid etching, piranha solution (a mixture of 98% sulfuric acid and 30% hydrogen peroxide), or alumina-particle air abrasion followed by adhesive application enhanced bond strength between PEEK and resin, with sulfuric acid etching yielding the highest bond strengths. However, another study^22^ showed that the tested surface treatments, 98% sulfuric acid for 5 s, 30 s, and 60 s, or air abrasion with 45-μm aluminum oxide, or tribochemical silica coating with 110-µm grain size (silica coating by air abrasion with silica-coated alumina particles), did not lead to a significant increase in resin bond strength to PEEK; pre-test failures occurred with all such pretreatments.^22^ It must also be considered that although some studies indicate that sulfuric acid is the best choice for treating PEEK,^28,41^ its toxicity and corrosive risks have made this material unsuitable for clinical use, or at least very challenging to use clinically.

It is important to highlight that the chemical interactions involving different bonding agents appear to play a relevant role in bond enhancement to PEEK. Several approaches have been evaluated,^6,16,17^ including the use of one- or two-step adhesives, silane bonding agents, or specific adhesives developed for composites and plastics. Stawarczyk et al^33^ reported that after alumina-particle air abrasion, the use of one-step adhesives (Visio.link, Bredent, Senden, Germany; Scotchbond Universal, 3M Oral Care, St Paul, MN, USA; Dialog bonding, Schütz-Dental, Rosbach, Germany) showed higher bond strengths between PEEK and veneering resin composite, while the combination of a primer (Monobond plus, Ivoclar Vivadent; Schaan, Liechtenstein) and an adhesive (Heliobond, Ivoclar Vivadent) resulted in lower bond strengths. However, another study indicated that when helium plasma was applied after aging by thermocycling, only Visio.link (Bredent) was able to promote increased bonding between PEEK and resin cements.^26^

Based on the assumptions above, the importance of acquiring high, stable bond strength between PEEK and resin materials is clear, but there is to date no consensus on the best option to achieve this end. A scoping review may help to elucidate this topic, discuss it, and to compile information to guide clinical decision making on which surface treatment to execute and which adhesive to apply when using PEEK-based restorative set-ups. Thus, through a scoping review, the goal of this study was to identify and discuss the available surface treatments and bonding agents for PEEK to increase its bond strength to resin-based materials used in dentistry.

## Material and Methods

### Protocol and Registration

The protocol of this study was prospectively based on the framework proposed by Peters et al,^20^ according to The Joana Briggs Institute, and is available for the public on the Open Science Framework platform (https://osf.io/4nur9/). The reporting of this scoping review was based on PRISMA Extension for Scoping Reviews.^34^

### Eligibility Criteria

We selected studies that evaluated and discussed PEEK surface treatment protocols, the use of adhesives, and the achieved bond strength to resin-based dental materials. We did not restrict the studies by year or language of publication: study designs which did not provide assessment of the desired outcome (ie, bond strength) were excluded. The corresponding authors of all studies which were not available for full-text reading or were missing information, were contacted in three attempts by e-mail or any other source that allowed direct contact with the authors (social media platforms such as ResearchGate, Twitter, or Linkedin). The study was excluded if there was no response.

### Search

The search was last performed on January 10th, 2021, considering four databases: MEDLINE via PubMed, Scopus, Web of Science, and Cochrane. The search strategy (Table 1) was based on MESH terms and free-text specific terms of PubMed, which were then adapted for the other databases, if necessary.

**Table 1:** Search strategy used in each database

Database	Search strategy
PubMed	((polyetheretherketone[Text Word]) OR (PEEK[Text Word]) OR (PEEK) OR (polyether*[Text Word]) OR (polyether ether ketone[MeSH Terms]) OR (polyether ether ketone[Text Word]) OR (Polyether ketone[MeSH Terms]) OR (Polyether ketone[Text Word])) AND ((abrasion, dental air[MeSH Terms]) OR (aluminum oxide[MeSH Terms]) OR (silicon dioxide[MeSH Terms]) OR (sulfur acids[MeSH Terms]) OR (hydrofluoric acid[MeSH Terms]) OR (fluorhydric acid[MeSH Terms]) OR (abrasion, dental air[Text Word]) OR (aluminum oxide[Text Word]) OR (silicon dioxide[Text Word]) OR (acid etching[Text Word]) OR (sulfur acids[Text Word]) OR (hydrofluoric acid[Text Word]) OR (fluorhydric acid[Text Word]) OR (sandblast*[Text Word]) OR (cement*[MeSH Terms]) OR (luting agent*[MeSH Terms]) OR (surface treatment[Text Word]) OR (Cojet[Text Word]) OR (laser[Text Word]) OR (resin cement*[MeSH Terms]) OR (resin cement*[Text Word]) OR (Silica coating[Text Word]) OR (laser[Text Word]) OR (argon plasma[Text Word]) OR (bond*[Text Word]) OR (jet[Text Word]) OR (adhesive* [text word])) AND ((“Bond strength”[MeSH Terms]) OR (“Bond strength”[Text Word]) OR (Bonding[Text Word]) OR (shear[MeSH Terms]) OR (shear[Text Word]) OR (tensile[MeSH Terms]) OR (tensile[Text Word]) OR (“push out”[MeSH Terms]) OR (“push out”[Text Word]) OR (“pull out”[MeSH Terms]) OR (“pull out”[Text Word]) OR (adhesion[MeSH Terms]) OR (adhesion[Text Word]))
Scopus	TITLE-ABS-KEY (polyetheretherketone OR peek OR (peek) OR polyether OR “polyether ether ketone” OR “polyether ether ketone” OR “Polyether ketone”) AND TITLE-ABS-KEY (“abrasion, dental air” OR “aluminum oxide” OR “silicon dioxide” OR “sulfur acids” OR “hydrofluoric acid” OR “fluorhydric acid” OR “acid etching” OR “sulfur acids” OR “sandblast” OR “cement” OR “luting agent” OR “surface treatment” OR “Cojet” OR “laser” OR “resin cement” OR “Silica coating” OR laser OR “argon plasma” OR bond OR jet OR adhesive) AND TITLE-ABS-KEY (“Bond strength” OR bonding OR shear OR tensile OR “Push out” OR “Pull out” OR adhesion)
Web of Sciences	TS=(polyetheretherketone OR PEEK OR PEEK OR “polyether ether ketone” OR “Polyether ketone” OR polyetherk*) AND TS=(“abrasion, dental air” OR “aluminum oxide” OR “silicon dioxide” OR “sulfur acids” OR “hydrofluoric acid” OR “fluorohydric acid” OR “acid etching” OR sandblast OR cement OR “luting agent” OR “Surface treatment” OR Cojet OR laser OR “resin cement” OR “Silica coating” OR “argon plasma” OR bond OR jet OR adhesive) AND TS=(“Bond strength” OR Bonding OR Shear OR Tensile OR “Push out” OR “Pull out” OR Adhesion)
Cochrane	((polyetheretherketone[Text Word]) OR (PEEK[Text Word]) OR (PEEK) OR (polyether*[Text Word]) OR (polyether ether ketone[MeSH Terms]) OR (polyether ether ketone[Text Word]) OR (Polyether ketone[MeSH Terms]) OR (Polyether ketone[Text Word])) AND ((abrasion, dental air[MeSH Terms]) OR (aluminum oxide[MeSH Terms]) OR (silicon dioxide[MeSH Terms]) OR (sulfur acids[MeSH Terms]) OR (hydrofluoric acid[MeSH Terms]) OR (fluorhydric acid[MeSH Terms]) OR (abrasion, dental air[Text Word]) OR (aluminum oxide[Text Word]) OR (silicon dioxide[Text Word]) OR (acid etching[Text Word]) OR (sulfur acids[Text Word]) OR (hydrofluoric acid[Text Word]) OR (fluorhydric acid[Text Word]) OR (sandblast*[Text Word]) OR (cement*[MeSH Terms]) OR (luting agent*[MeSH Terms]) OR (surface treatment[Text Word]) OR (Cojet[Text Word]) OR (laser[Text Word]) OR (resin cement*[MeSH Terms]) OR (resin cement*[Text Word]) OR (Silica coating[Text Word]) OR (laser[Text Word]) OR (argon plasma[Text Word]) OR (bond*[Text Word]) OR (jet[Text Word]) OR (adhesive* [text word])) AND ((“Bond strength”[MeSH Terms]) OR (“Bond strength”[Text Word]) OR (Bonding[Text Word]) OR (shear[MeSH Terms]) OR (shear[Text Word]) OR (tensile[MeSH Terms]) OR (tensile[Text Word]) OR (“push out”[MeSH Terms]) OR (“push out”[Text Word]) OR (“pull out”[MeSH Terms]) OR (“pull out”[Text Word]) OR (adhesion[MeSH Terms]) OR (adhesion[Text Word]))


### Screening Protocol

The search was initially performed using the Rayyan program. Three independent researchers identified articles by first analyzing titles and abstracts for relevance and the presence of the eligibility criteria. Retrieved records were classified as included, excluded, or uncertain. The full-text articles of the included and uncertain records were selected for further eligibility screening by the same three reviewers. Discrepancies in screening of titles/abstracts and full text articles were resolved through a discussion between the researchers. In case of disagreement, the opinion of a fourth researcher was requested.

### Charting the Results

We created a form using Microsoft Excel, which was tested by three reviewers to reach a consensus on data collection. Then, two reviewers independently extracted the data and another reviewer checked it. The following data were collected according to each study type included. For reviews, this included author, year of publication, study design, data base considered, eligibility criteria, number of included studies, quality of evidence reported by the authors, main findings. For in vitro studies, this comprised title, authors, data of publication, PEEK characteristics, PEEK surface treatments (with protocols of use), control group (presence or absence), bonded set, luting agent, specimen geometry, storage (if present), thermocycling (if present), pre-test failures (if present), bonding test, test geometry, failure analysis, main findings, and the compliance of the executed test with normative guidelines.

### Data Analysis

A descriptive analysis was performed considering the extracted data using tables and figures, and sub-grouped by the study design (reviews or in vitro studies).

## Results

**Fig 1:**
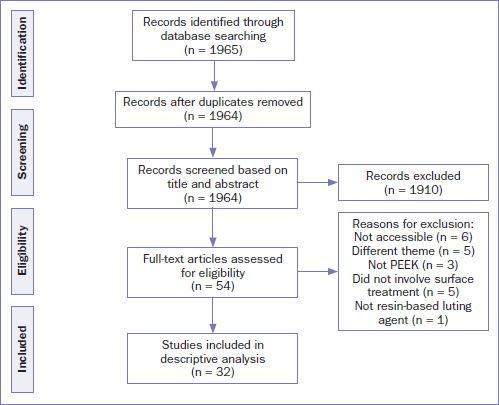
Flowchart of the study search and screening.

The search and screening protocol are illustrated in a PRISMA flowchart (Fig 1). From a total of 1965 studies selected by the search strategy, 54 were considered eligible for full-text screening, and 32 studies (3 reviews and 29 in vitro) were included for qualitative analysis. The main characteristics of the included studies are described in Table 2 (reviews) and 3 (in vitro).

**Table 2:** Descriptive data for the included reviews

Author and year	Study design	Database considered	Eligibility criteria	Number of included studies	Quality/level of evidence reported by the authors	Main findings
Alexakou et al, 2019	Narrative review	PubMed	Not reported	Not reported	Not reported	There was no agreement on a common preparation protocol of PEEK surface.
Escobar et al, 2019	Short review	Medline	Published in vitro, meta-analysis; prospective cohort studies, from 1995 to 2019, written in English, and exploring PEEK surface treatments and the achieved adhesion to resin-matrix composites.	10 studies	Not reported	Sulfuric acid etching and air abrasion treatments promoted an increase in the adhesion of PEEK to resin-matrix composites. Methacrylate-based adhesives also enhanced the adhesion of PEEK to resin-matrix composites.
Gama et al, 2020	Systematic review	Embase; Latin American and Caribbean Health Sciences; PubMed; SCOPUS; Web of Science; Google Scholar; Open Grey; ProQuest	Studies evaluating the ability of surface treatments to improve high-performance polymer bond strength for dental applications were included. No publication time or language restrictions were applied, the presence of control group was mandatory, subgroups with fewer than 5 specimens were excluded.	11 studies	Strong; low risk of bias	The use of surface treatments in association with a bonding system is important to increase the bond strength between high performance polymers and veneering resin composites, even when aging protocols (water storage and/or thermocycling) were performed. Of the various mechanical and chemical pretreatments employed, only air abrasion was applied with similar methodologies between studies, thus allowing metanalysis. The application of air abrasion (50-μm alumina) improved the bond strength. Likewise, silica-coated air abrasion increased the bond strength.


Considering the data compiled by the three reviews (Table 2), it can be observed that Alexakou et al^1^ gathered existing data using a narrative review on this theme, but it did not report much data that would allow accessing the method used to compile such information, the accuracy of the information, or the quality/level of evidence of the findings. Furthermore, those authors inferred the importance of executing surface treatments on a PEEK surface, but without providing any protocol to do so. Escobar et al^9^ performed a short review, which is an important method to rapidly access the literature to obtain information on a theme, but it used simplified methods which may not guarantee the inclusion of all existing information on a given theme, and in doing so, it decreases its reliability and quality/level of evidence (which is not reported by the authors). Despite its limitations, Escobar et al^9^ points to the use of sulfuric acid or air-abrasion protocols as viable treatments to enhance adhesion primarily based on 10 included studies. Finally, Gama et al^11^ compiled information about adhesion to high performance polymers using a systematic review, also considering PEEK. They used a very strict method and therefore only a few studies were included (11 studies, 8 of which addressed PEEK). In addition, heterogeneous substrates (many types of high-performance polymers) were included and surface treatments with different parameters were evaluated. Based on that, although those authors reported a strong level of evidence, the discussion and comparison between conditions (different materials and protocols) was very limited.^11^ As conclusions, Gama et al^11^ emphasized the need of executing surface treatments and advocated the use of air-abrasion protocols (silica coated or not) to increase bond strength between the polymers and resin-based composites.

The 29 in vitro studies included in the present scoping review (Table 3) evaluated various PEEK compositions, including unfilled PEEK (12 studies), PEEK reinforced with 20 wt% titanium oxide (17 studies), PEEK reinforced with 20 wt% titanium oxide and 1% pigment (2 studies), glass fiber-reinforced PEEK (4 studies), and carbon fiber-reinforced PEEK (1 study). However, only 3 studies evaluated the effect of surface treatments under different PEEK materials, showing that when different PEEK compositions are considered, they may result in more challenging scenarios to enhance adhesion, as seen by Yan et al^38^ with unfilled PEEK. Nevertheless, consensus seems to exist that surface treatments may increase the bond strength for all PEEK materials.

**Table 3:** Descriptive data for the included in vitro studies

Authors (year)	PEEK characteristics	Bonded set	Test geometry	Control condition	Surface treatments	Aging protocol	Main findings
Ates et al, 2018	PEEK reinforced with 20% weight titanium oxide (breCAM.BIOHPP, Bredent)	PEEK + adhesive (Visio.link, Bredent)+ flowable opaque composite (Crea.lign opaker A2, Bredent) + veneering composite resin (Crea.lign paste A2, Bredent).	Shear bond strength (knife-edge) Cylinder dimensions: diameter: 4 mm; height: 5 mm	Untreated condition	Air abrasion with alumina (50-µm grain size, 15 s); Air abrasion with silica coated (30-µm grain size, 15 s); Er:YAG laser: 2.940 nm, 150 mJ, 10 Hz, 1.5 Win QSP mode; Er:YAG laser + air abrasion with alumina (50-µm grain size, 15 s); Er:YAG laser + air abrasion with silica-coated alumina particles (30-µm grain size, 15 s).	Storage: 24 h in water at 37ºC; +thermocycling: 5000 cycles at 5°C to 55°C with a dwell time of 20 s.	Air abrasion with alumina and silica-coated alumina particles alone or combined with use of Er:YAG laser promoted the highest bond strength, followed by the application of laser alone and no treatment, which promoted lower values.
Bötel et al, 2018	Different types of PEEK: unfilled (Juvora Dental disk, Invibio); white filled with 20% titanium oxide powder (TiO_2_) (DC4420, Daicel, EVONIK); filled with 20% TiO_2_ powder and 1% pigment (DC4450, Daicel, EVONIK)	PEEK + adhesive (Visio.link, Bredent) + veneering composite resins (Vita VM LC, VITA; or GC GRADIA, GC; or GC GRADIA DIRECT Flo [GC])	Shear bond strength Cylinder dimensions: diameter: 5 mm; height: 2 mm Approach unclear*	Air abrasion only	All specimens air abraded with alumina (110-µm grain size, 10 s) followed by ultrasonic bath and low-pressure plasma treatments: O_2_ plasma (3 min); O_2_ plasma (35 min); Argon/ O_2_ plasma (3 min); Argon/ O_2_ plasma (35 min). Constant parameters between the groups (temperature = 70°C; pressure = 0.3 mbar; frequency = 100 kHz; energy = 200W).	Storage: 24 h in water at 37ºC.	The surface pretreatment of diverse PEEK types with low-pressure plasma prior to veneering with composite generated a positive influence on the adhesive bond between PEEK and composite. Plasma with oxygen process gas for a duration of 35 min and the use of low viscosity cement (Gradia Direct Flo) promoted the highest bond strength.
Caglar et al, 2018	PEEK reinforced with 20wt% titanium oxide (breCAM.BIOHPP, Bredent)	PEEK + adhesives (none; or Visio.link, Bredent; or Signum PEEK bond, Heraeus Kulzer) + resin cement (Panavia SA Cement Plus, Kuraray)	Shear bond strength (knife edge) Cylinder dimensions: diameter: 4 mm, height: 5 mm	Untreated condition	Air abrasion with alumina (50-µm grain size, 15 s); air abrasion with silica-coated alumina particles (30-µm grain size, 15 s); Er:YAG laser: 2.940 nm, 150 mJ, 10 Hz, 1.5 W in QSP mode.	Storage: 24 h in water at 37ºC; + thermocycling: 5000 cycles at 5 to 55°C with a dwell time of 20 s.	All groups presented lowest values when the adhesive was not applied. Both air abrasion groups with alumina and silica-coated alumina particles, mainly associated with one-step universal adhesive (Visio.link), promoted higher bond strength, followed by Er:YAG laser and no treatment, which presented lower values. The one-step universal adhesive (Visio.link) promoted the highest values compared with two-step adhesive (Signum PEEK bond).
Chaijareenont et al, 2018	PEEK reinforced with titanium dioxide pigments and 20% ceramic-filled (Dentokeep, Nt-trading)	PEEK + adhesive (Heliobond; Ivoclar Vivadent) + veneering composite resin (Filtek Z350XT, 3M Oral Care)	Shear bond strength Cylinder dimensions: diameter: 3 mm; height: 2 mm Approach unclear*	Untreated condition	Different percentages of sulfuric acid: 70%, 80%, 85%, 90%, and 98% for 60 s.	Storage: 24 h in water at 37ºC.	Concentrations of 85%, 90% and 98% promoted the highest bond strength. 80% promoted intermediate strength and statistically similar to the strength with 85%. The 70% and no treatment presented the lowest values.
Çulhaoğlu et al, 2017	PEEK reinforced with 20% weight titanium oxide (breCAM.BIOHPP, Bredent)	PEEK + adhesive (Visio.link, Bredent) + veneering composite resin (Combo.lign, Bredent)	Shear bond strength Cylinder dimensions: diameter: 2 mm; height: 2 mm Approach unclear*	Untreated condition	Air abrasion with silica-coated alumina particles (30-µm grain size, 15 s); acetone 99% (60 s); 98% sulfuric acid (60 s); air abrasion with alumina (110-µm grain size, 15 s); Yb:PL laser: 5 W, 250 ms frequency.	Storage: not mentioned Thermocycling: 10,000 cycles at 5 to 55°C with a dwell time of 20 s.	Sulfuric acid promoted the highest bond strength. Laser, air abrasion with 30-μm silica-coated alumina and 110-μm alumina particles promoted intermediary adhesion. Acetone and no treatment promoted the lowest bond strength.
Fuhrmann et al, 2014	Glass fiber-reinforced PEEK (Cendres+Métau)	PEEK or PEKK (amorph) or PEKK (crystalline) Silane (Monobond Plus, Ivoclar Vivadent) and/or adhesive primer (Luxatemp Glaze & Bond, DMG) + resin cement (Multilink Automix, Ivoclar Vivadent) + luting composite (Multicore Flow, Ivoclar Vivadent)	Tensile bond strength Cylinder diameter: 3.2 mm Approach: Specimen positioned and aligned in a jig. The jig was attached to the load cell crosshead by upper and lower chains, allowing the whole system to be self-aligning and providing a moment-free axial force application (crosshead speed: 2 mm/min).	Negative control: air abrasion with alumina particles (110-µm grain size, 15 s) + resin cement (Multilink Automix), without adhesive; positive control: Air abrasion with alumina particles (110-µm grain, 15 s) + adhesive primer (Luxatemp Glaze & Bond) 20 s + resin cement (Multilink Automix)	Air abrasion with alumina (110-µm grain, 15 s) + adhesive primer (Luxatemp Glaze & Bond) with storage under a lightproof box for 5 min + resin cement (Multilink Automix); air abrasion with silica-coated alumina particles (30-µm grain size, 15 s) + silane (Monobond Plus) for 5 min + resin cement (Multilink Automix); air abrasion with silica-coated alumina particles (30-µm grain size, 15 s) + silane (Monobond Plus) + adhesive primer (Luxatemp Glaze) + resin cement (Multilink Automix)	Storage: water at 37ºC for 3, 30 and 150 days + thermocycling: 10,000 (30 days) or 37,500 (150 days) cycles at 5 to 55°C.	Highest bond strength with the combination of 30-µm silica-coated alumina particles associated, universal primer (Monobond Plus) and resin primer (Luxatemp Glaze Bond). The fiber-reinforced PEEK material provided the highest TBS of all three materials within all conditioning groups. Although tensile bond strength decreased slightly over the storage time of 150 days, these decreases were not statistically significant in most subgroups.
Hallmann et al, 2012	PEEK dental-based polymer (PEEK-OPTIMA, Invibio Biomaterial Solutions)	PEEK + adhesives (Heliobond, Ivoclar Vivadent; or Clearfil Ceramic Primer, Kuraray) + resin cement (RelyX Unicem, 3M Oral Care)	Tensile bond strength Cylinder diameter: 3 mm Approach unclear*	Untreated condition	Piranha solution, 30 s; air abrasion with alumina (50-µm grain size, 15 s) + piranha solution, 30 s; air abrasion with alumina (110-µm grain size, 15 s) + piranha solution, 30 s; air abrasion with silica-coated alumina particles (30-µm grain size) + piranha solution, 30 s; air abrasion with silica-coated alumina particles (110-µm grain size) + piranha solution, 30 s	Storage: 3 days in water at 37ºC.	It was not possible to obtain good adhesion between the resin cement and the non-treated surface. Combination with air abrasion and piranha solution promoted higher bond strength for both adhesives.
Henriques et al, 2018	Different types of PEEK: Conventional unfilled PEEK (TECAPEEK, Ensinger); PEEK reinforced with 30vol% glass fiber (TECAPEEK, Ensinger); PEEK reinforced with 30vol% carbon fiber (TECAPEEK, Ensinger)	PEEK + resin cement (Allcem CORE, FGM)	Shear bond strength Specimen dimension unclear* Approach unclear*	98% sulfuric acid as control	CO_2_ laser: 200 μm holes spaced 400 μm apart; CO_2_ laser: 200 μm holes spaced 600 μm apart; CO_2_ laser: 200 μm holes spaced 400 μm apart + 98% sulfuric acid. Laser parameters: wavelength 1064 nm, 50 W, input power 1000 W, 50 Hz.	Storage: distilled water at 37°C for 24 h.	For unfilled PEEK, acid promoted highest bond strength. For carbon modified PEEK, the samples from laser treated showed a significant increase in the bond strength compared with the traditional acid etching treatment. The acid-etched samples had a substantial reduction in the bond strength comparing with the unfilled and glass-reinforced PEEK samples. Samples treated with both acid etching and laser promoted the lowest values.
Jahandideh et al, 2020	PEEK reinforced with 20% weight titanium oxide (Bio.HPP, Bredent)	PEEK + adhesive (Visio.link, Bredent) + resin Opaquer (Crea.Lign Opaquer, Bredent) + veneering composite resin (Crea.lign paste A2, Bredent).	Shear bond strength Cylinder dimensions: diameter: 4 mm; height: 1.5 mm; Approach unclear*	Untreated condition	CO_2_ laser with 4 W power, 159.22 J/cm^2^, wavelength of 10600 nm, for 50 s; Er:YAG laser with a 2940 nm wavelength, 150 mJ energy, for 20 s, 1.5 W output power and 119.42 J/cm^2^ energy density in a pulse mode (10 Hz) with 700 μs pulse duration at a 10 mm.	Storage: distilled water at 37°C for 24 h.	Er:YAG laser promoted the highest bond strength. The CO_2_ laser was promoted intermediate strength and the control group promoted the lowest bond strength.
Keul et al, 2014	PEEK reinforced with 20% of inorganic filler (Dentokeep, Nt-trading)	PEEK + silane and/or adhesive (no adhesive; or Monobond Plus/ Heliobond, Ivoclar Vivadent; or Visio.link, Bredent; or Clearfil Ceramic Primer, Kuraray; or Signum PEEK Bond I+II, Heraeus Kulzer) + veneering composite resins (Signum Composite Dentin shade A3, Heraeus Kulzer; or Signum Ceramis Dentin shade A3, Heraeus Kulzer).	Tensile bond strength Cylinder dimensions: diameter: 2.9 mm; height: 6 mm Approach: Specimens positioned in a jig with the specimen’s surface perpendicular to the loading direction. The jig was attached to the load cell and pulled apart by an upper chain. Crosshead speed of 5 mm/min.	Untreated condition	Air abrasion with alumina particles (50-µm grain size, 10 s); piranha solution (30 s) Air abrasion + piranha solution (30 s).	Storage: distilled water at 37°C for 60 days + thermocycling: 5000 cycles between 5°C and 55°C with a dwell time of 20 s.	Air abrasion alone or combined with piranha solution promoted the highest bond strength, mainly associated with universal one-step adhesive (Visio.link), and two-step adhesives (Signum PEEK Bond and Monobond Plus/ Heliobond). The use of adhesive containing 10-methacryloyldecyl dihydrogen phosphate-MDP (Clearfil ceramic Primer) showed no or low bond strength.
Lümkemann et al, 2018	PEEK reinforced with 20% of inorganic filler (Dentokeep, Nt-trading)	PEEK + adhesives (none; or Scotchbond Universal, 3M Oral Care; or Clearfil Universal Bond, Kuraray; or Futurabond U, Voco; or Adhese Universal, Ivoclar Vivadent; or G-Premio Bond, GC; or Pekk Bond, Anaxdent; or Visio.link, Bredent) + resin cement (Clearfil SA, Kuraray)	Tensile bond strength; Cylinder diameter: 2.9 mm Approach: The specimens were positioned in a jig with the PEEK surface perpendicular to the load. Crosshead speed of 5 mm/min.	Negative control: untreated Positive control: Visio.link	Air abrasion with alumina particles (50-µm grain size, 10 s), using different pressures: 0.05 MPa; 0.2 MPa; 0.4 MPa.	Storage: distilled water at 37°C for 24 h; + thermocycling: 5000 cycles between 5°C and 55°C with a dwell time of 20 s.	Air abrasion pressure does not have an impact on the results of the tensile bond strength, except with adhesives containing methyl methacrylate- MMA and urethane dimethacrlyate (UDMA) (Pekk Bond). Universal adhesives (Scotchbond Universal and Adhese Universal) were comparable to one-step control adhesive (Visio.link).
Rocha et al, 2016	Conventional unfilled PEEK (Juvora Dental Disk, Juvora)	Dentin + two successive layers of adhesive over dentin (Adper Single Bond 2, FGM) + resin cement (RelyX, ARC, 3M Oral Care) + silane agent (RelyX Ceramic Primer, 3M Oral Care) over PEEK + PEEK	Shear bond strength Cylinder dimensions: diameter: 3 mm; height: 3 mm Approach: The bonding interface of PEEK and dentin was positioned perpendicular to the horizontal plane and a stainless-steel wire (diameter: 0.4 mm) applied shear force at the interface with a crosshead speed of 0.5 mm/min until fracture.	Not mentioned	Air abrasion with alumina particles (45 μm grain size, 15 s) Air abrasion with alumina particles (45 μm grain size) + silica coated alumina particles (110-µm grain, both for 15 s); 98% sulfuric acid (5 s); 98% sulfuric acid (30 s); 98% sulfuric acid (60 s).	Storage: distilled water at 37°C for 24 h.	No differences between experimental groups. Adhesive failures predominant.
Rosentritt et al, 2014	Conventional unfilled PEEK (Juvora Dental Disk, Juvora)	PEEK + adhesives (none; Espe Sil, 3M Oral Care; or Signum Conector, Heraeus Kulzer; or Solidex Solibond, Shofu; or Composite primer, GC; or New Outline Primer, Anaxblend; or Clearfill Aloy Primer, Kuraray; or Clearfill Ceramic Primer, Kuraray; or New Outline, Anaxblend; or Metal Bonder, Anaxblend; or Cera Resin Bond, Shofu; or ML Primer, Shofu; or Metal Primer II, GC; or Plaquit, Dreve; or Zirconia Bond, Heraeus Kulzer) + opaque (None; Opaque; Flowapaque; Clearfil Opaque) + veneering composite resin (Sinfony, 3M Oral Care; or Signum, Heraeus Kulzer; or Solidex, Shofu; or Gradia, GC; or Anaxblend, Anaxblend; or GC Gradia, GC)	Shear bond strength Cylinder dimensions: diameter: 5 mm; height: 4 mm Approach unclear*	Not mentioned	98% Sulfuric acid (60 s); piranha solution (1:1 30 s); air abrasion with alumina particles (50-μm/2 bar); air abrasion with alumina particles (120-μm/2.8 bar); air abrasion with silica-coated alumina particles (30-μm/110-μm).	Storage: distilled water at 37°C for 24 h; or distilled water at 37°C for 90 days or thermocycling; or thermocycling: 12,000 cycles at 5°C and 55 °C.	For achieving good bonding between PEEK and veneering composite, surface cleaning and roughening (air abrasion, tribochemical treatment, sulfuric acid) is recommended. For adhesive bonding, a surface treatment prior to bonding seems to be essential. The combination with the application of opaque materials in most cases revealed an increase in shear bond strength. The data showed that seven of the tested systems mainly including phosphate components achieved sufficient shear bond strength. The combination of surface roughening and subsequent activation with acetone- or phosphate-based methacrylate primers or tribochemical treatment yields highest bond strengths on PEEK surfaces. For most systems, no significant changes were found in comparison to the baseline data.
Schmidlin et al, 2010	Conventional PEEK (PEEK-CLASSIX)	PEEK + adhesive (None; Heliobond, Ivoclar Vivadent) + resin cements (RelyX Unicem, 3M Oral Care; or Tetric, Ivoclar Vivadent)	Shear bond strength Cylinder diameter: 3.1 mm Approach: The specimens were positioned in the sample holder and parallel to the loading piston in a distance of 200 μm. The loading piston had a chisel configuration and load was applied with a crosshead speed of 1 mm/min.	Titanium material was treated as material control. Untreated condition for PEEK control group	98% sulfuric acid (1 min) Air abrasion with alumina particles (50-µm grain size, 10 s Air abrasion with alumina particles (110-µm grain, 10 s) Air abrasion with alumina particles (110-µm grain, 10 s) + air abrasion with silica-coated alumina particles (110-µm grain, 12 s) + application of ESPE Sil (3M Oral Care) and air drying for 5 min.	Storage: distilled water at 37°C for 24 h.	On the polished surfaces, no adhesion could be established with either resin system. Air abrasion with 50- or 110-μm alumina, or silica-coated alumina particles, did not lead to any PEEK adhesion with the universal composite resin cement (RelyX Unicem). Higher bond strengths were obtained by 98% sulfuric acid groups, for both universal and two-step system (Heliobond/ Tetric).
Schmidlin et al, 2016	PEEK reinforced with 20% of inorganic filler (Dentokeep, Nt-trading)	PEEK + adhesives (None; or Soft-Liner Liquid, GC; or Visio.link, Bredent; or Ambarino P60, Creamed) + glycine (half of specimens) + resin cements (RelyX Unicem, 3M Oral Care; or Clearfil SA Cement, Kuraray)	Tensile bond strength Cylinder diameter: 2.9 mm Approach Unclear*	Air abrasion only	For all specimens: Air abrasion with alumina particles (50-µm grain size, 10 s) Low-density cold helium plasma for 20 s with pressure of 0.2 MPa at distance of 10 mm.	Storage: distilled water at 37°C for 24 h and half of the specimens were additionally aged for another 14 days + thermocycling: 10,000 cycles at 5°C and 55°C with a dwell time of 20 s.	The one-step universal adhesive (Visio.link) showed the highest tensile bond strength (TBS) in all tested groups. The other adhesives and no-adhesive group showed no bond (0 MPa), unless with a combination of argon plasma and glycine application. The aging process decreased the bond strength for groups with application of plasma and glycine.
Schwitalla et al, 2017	Unfilled PEEK type (Juvora dental disk, Juvora); PEEK containing 20% titanium oxide (TiO_2_) powder (Vestakeep DC4420, Evonik); colored PEEK compound filled with 20% TiO_2_ powder and about 1% of pigment powder (Vestakeep DC4450, Evonik)	PEEK + adhesive (Visio.link, Bredent) + resin cement (Vita VM LC, VITA)	Shear bond strength Cylinder dimensions: diameter: 5 mm; height: 2 mm Approach unclear*	Untreated condition	Plasma treatment (35 min at 70 ºC and a pressure of 0.3 bar with a frequency of 100 kHz and a power output of 200W) Air abrasion with alumina particles (110-µm grain, 10 s) Air abrasion with alumina particles (110-µm grain, 10 s) and plasma treatment.	Storage: distilled water at 37°C for 24 h.	Alumina air abrasion + plasma of Juvora exhibited the highest bond strength. The shear bond strength for all PEEK types increased for all treated groups, except for Juvora PEEK, where control and plasma were similar. The air-abraded specimens displayed a significant increase in adhesion in comparison to the equivalent polished specimens.
Silthampitag et al, 2016	PEEK reinforced with titanium dioxide pigments, ceramic-filled 20% (Dentokeep, Nt-trading)	PEEK + adhesives (mone; or Heliobond, Ivoclar Vivadent) + veneering composite resin (Filtek Z350XT Flowable, 3M Oral Care)	Shear bond strength Cylinder dimensions: diameter: 3 mm, height: 2 mm Approach: The specimen was fixed in a special jig to align a chisel-shaped rod parallel to the bond surface at the bonding interface, at a crosshead speed of 1 mm/min.	Untreated condition	98% sulfuric acid (60 s); piranha solution: a mixture of 98% sulfuric acid and 30% hydrogen peroxide in a ratio 10:3 for 30 s; air abrasion with alumina particles (50-µm grain size, 10 s).	Storage: distilled water at 37°C for 24 h.	The specimens in the 98% sulfuric acid and the use of adhesive showed the highest shear bond strength (SBS). Control and piranha solution showed the lowest bond strength without adhesive.
Sproesser et al, 2014	PEEK reinforced with 20% of inorganic filler (Dentokeep, Nt-trading)	PEEK + resin cements (RelyX ARC, 3M Oral Care; or Variolink II, Ivoclar Vivadent; or Clearfil SA Cement, Kuraray).	Shear bond strength Cylinder diameter: 2.9 mm Approach unclear*	Untreated condition	Different etching times of 98% sulfuric acid (20 μl): 5 s; 15 s; 30 s; 60 s; 90 s; 120 s; or 300 s.	Storage: distilled water at 37°C for 28 days.	Regardless of the etching duration, the self-adhesive resin cement (Clearfil SA) showed lower bond strength values than conventional resin composites (RelyX ARC or Variolink II). Control group showed no bond. For all cements, intermediate acid etching generated higher bond strengths. More than 120 s produced negative effects.
Stawarczyk et al, 2013 A	PEEK reinforced with 20% of inorganic filler (Dentokeep, Nt-trading)	PEEK + veneering composite resin (Gradia, GC; or Sinfony, 3M Oral Care)	Shear bond strength Cylinder dimensions: diameter: 2.9 mm, thickness: 1.5 mm Approach unclear*	Untreated condition	98% sulfuric acid (60 s); Air abrasion with alumina particles (50-µm grain size, 10 s); Air abrasion with alumina particles (110-µm grain size, 10 s); Air abrasion with alumina particles (110-µm grain size, 10 s) + air abrasion with silica-coated alumina particles (110-µm grain size, 12 s), and ESPE Sil, 5 min.	Storage: 3 days in distilled water at 37ºC.	Both veneering composite resins showed higher shear bond strength after acid etching. The lowest values were observed for untreated and 50-μm air-abraded groups.
Stawarczyk et al, 2013 B	PEEK reinforced with 20% of inorganic filler (Dentokeep, Nt-trading)	PEEK + adhesives (None; Visio.link, Bredent; or Signum PEEK Bond I + II, Heraeus Kulzer; or Ambarino P60, Creamed) + resin cements (RelyX Unicem Automix 2, 3M Oral Care; or Clearfil SA, Kuraray)	Shear bond strength Cylinder dimensions: diameter: 2.9 mm, thickness: 1.5 mm Approach unclear*	Untreated condition	Plasma pretreatment (20 s, 200 kPa, 10 mm of distance).	Storage: 24 h in water at 37ºC; + thermocycling: 5000 or 10,000 cycles.	The plasma treatment and the choice of resin cement had no impact on the shear bond strength. The use of adhesive affected positively the bond strength. Plasma application without adhesive showed no bonding to PEEK with both tested self-adhesive resin cements. The one-step (Visio.link) and two-step adhesive (Signum PEEK Bond) on surfaces treated with and without plasma showed higher bond strengths. The one-step primer (Ambarino P60) revealed no bond. The 10,000 TC-aged plasma groups combined with Signum PEEK bond showed significantly lower SBS regardless of the resin cement used.
Stawarczyk et al, 2014	PEEK reinforced with 20% of inorganic filler (Dentokeep, Nt-trading)	PEEK + adhesives (None; or Visio.link, Bredent; or Signum PEEK Bond I + II, Heraeus Kulzer) + resin cements (Vita VM LC, VITA Zahnfabrik; or Sinfony, 3M Oral Care)	Tensile bond strength Cylinder dimensions: diameter: 2.9 mm; height: 10 mm Approach: Specimens positioned in a jig (perpendicular to the loading direction). The jig was attached to the load cell and pulled apart by an upper chain (crosshead speed of 5 mm/min).	Untreated condition	98% sulfuric acid (60 s); piranha solution (30 s).	Storage: half for 24 h in distilled water at 37ºC; and half in distilled water for 60 days at 37ºC.	The groups without use of adhesives showed the lowest tensile bond strength. Both adhesives were effective to promote adhesion. The pretreatments were similar for each adhesive, so there was no need for acid etching. The less viscous cement (Sinfony) showed higher bond strength. An increase as well as a decrease of TBS was observed between groups after 60 days of water storage at 37°C.
Stawarczyk et al, 2018	Conventional PEEK (Tizian PEEK, Schütz Dental)	PEEK + adhesives (Visio.link, Bredent; or Monobond Plus/Heliobond, Ivoclar Vivadent; or Scotchbond Universal, 3M Oral Care; or Dialog bonding fluid, Schütz Dental) + veneering composite resin (Dialogue Occlusal, Schütz Dental)	Tensile bond strength Cylinder dimensions: diameter: 2.9 mm; height: 10 mm Approach: specimens positioned in a holding device, which was attached to the load cell and pulled (crosshead speed of 5 mm/min).	Positive control: Visio.link (Bredent) adhesive groups	Air abrasion with 50-µm alumina particles with a pressure of 0.05 or 0.35 MPa Air abrasion with 110-µm alumina particles with a pressure of 0.05 or 0.35 MPa Air abrasion with 110-µm alumina modified with silica (0.28 MPa pressure).	Storage: 28 days in distilled water at 37°C; + thermocycling: 20,000 cycles, 5°C to 55°C, with a dwell time of 20 s.	The greatest influence on the bond strength was exerted by the use of an adhesive system followed by the pressure during air abrasion, while the grain size of the air-abrasion powder did not show any effect. The one-step control adhesive (Visio.link) showed the highest bond strengths, followed by a different one-step adhesive (Scotchbond Universal). The two-step adhesive showed the lowest bond strengths. Specimens air abraded with 0.35 MPa showed the highest survival rates.
Tsuka et al, 2017	PEEK reinforced with titanium dioxide (Vestakeep, Daicel- Evonik)	PEEK; or Dental gold-silver-palladium alloy; or zirconia; or hybrid composite resin + resin cements (Panavia V5, Kuraray; or RelyX Ultimate, 3M Oral Care; or G-CEM Link Force, GC; or Super-Bond C&B, Sun Medical)	Shear bond strength Cylinder diameter: 4 mm Approach: Specimens fixed with a special fixture (perpendicular to the loading direction). The loading piston was brought close to the bonding surface (crosshead speed of 1 mm/min).	Dental gold-silver-palladium alloy; zirconia; hybrid composite resin as control materials Untreated condition	Air abrasion with alumina particles (50-μm grain size, 10-mm distance, 10 s).	Storage: 24 h in distilled water at 37°C.	The PEEK group had a significantly lower shear bond strength compared with the control materials. In the PEEK group, the specimens using methyl methacrylate (MMA) cement (Super-Bond C&B) had higher bond strength compared to the other cements. Shear bond strengths for the air-abraded PEEK group were not significantly different than those for the PEEK group with no treatment.
Tsuka et al, 2018	PEEK reinforced with titanium dioxide (Vestakeep, Daicel- Evonik)	PEEK + resin cements (Panavia V5, Kuraray; or RelyX Ultimate, 3M Oral Care; or G-CEM Link Force, GC; or Super-Bond C&B, Sun Medical)	Shear bond strength Specimen dimensions unclear* Approach unclear*	Untreated condition	Air abrasion with alumina particles (50-µm grain size, 15 s); laser treatment 100 μm; laser treatment 150 μm; laser treatment 200 μm. Parameters of laser irradiation: irradiation speed of 500 mm/s, frequency of 25 kHz, exposure time of 33 s, pulse width of 8 ns, 197 mm from the surface, and a perpendicular angle of exposure.	Storage: 24 h in distilled water at 37°C.	The laser groove-treated specimens (100, 150, and 200 μm) had significantly higher shear bond strength than those in the other groups. On the other hand, in the laser groups there was no significant difference. When Panavia V5 was used, there was almost no adhesion in the no-treatment and air-abrasion treatment groups. The other cements promoted a positive effect on bond strength.
Wang et al, 2020	PEEK Unclear*	PEEK + Primer (Heliobond, Ivoclar Vivadent) + resin cement (Variolink, Ivoclar Vivadent)	Shear bond strength Cylinder dimensions: diameter: 3 mm; height: 4 mm Approach: unclear*	Untreated condition	Plasma treatment for 15, 25, or 30 min Frequency of 13.56 kHz and tension of 500 V.	Storage: One group for 56 h in distilled water at 37°C); or thermocycling for 5000 cycles or 10,000 cycles between 5°C and 55°C, 30 s each.	The shear bond strengths of the specimens were significantly higher in the treated groups than in the control group. The highest values were observed for 15 min of nitrogen plasma treatment (Pre-N25), followed by Pre-N35 and Pre-N15, respectively. The thermocycling decreased the SBS similarly for 5000 and 10,000 cycles.
Yan et al, 2013	PEEK resin; and 40 wt% barium-containing glass filler (BGF) reinforced PEEK (Changchun Jilin University Super Engineering Plastics Research Co.)	PEEK + resin cement (Dentex dual-cure resin cement, Sino-Dentex)	Shear bond strength Cylinder dimensions: diameter: 3.7 mm; length: 4 mm Approach: Specimen positioned in a holder (composite surface parallel to the load). The loading piston had an aperture configuration in which the cylinder could get through (crosshead speed of 0.7 mm/min) on the outer diameter of the cylinder in a distance of 200 mm from the composite surface.	Polishing with 600-grit silicon carbide papers as control group	9.5% hydrofluoric acid (2 min) Silica coating (dentex) 98% sulfuric acid (60 s) Polishing with 150-grit silicon carbide papers.	Storage: 24 h in distilled water at 37°C.	Most pristine PEEK specimens show failure after water storage, indicating its bond strength was nearly 0 MPa. After introduction of BGF particles, the bond strength between PEEK and resin cement were significantly increased. Polishing with 150-grit papers and sulfuric acid groups showed higher values of bond strength, with no statistical difference between them. Polishing with 600-grit papers, hydrofluoric acid and silica coating treatments showed lower bond strengths.
Younis et al, 2019	Unfilled PEEK (Optima PEEK, Juvora)	PEEK + adhesive only in Visio.link group (Visio.link, Bredent) + veneering composite resin (Sinfony, 3M Oral Care)	Shear bond strength Cylinder diameter: 5 mm Approach: Specimen positioned in a holder. A chisel-shaped rod applied force parallel to the bond surface at a distance of 0.5 mm from the surface of the PEEK specimen (1 mm/min crosshead speed.	Untreated condition	Plasma pretreatment with oxygen Plasma pretreatment with nitrogen Plasma pretreatment with argon Plasma pretreatment with air Vision.link adhesive only Parameters of plasma treatments: 10 min, 0.3 mbar, 20ºC, 40 kHz, power output of 100 W.	Storage: not mentioned Thermocycling: 5000 cycles between 5ºC and 55°C for 30 s in each, 20-s dwell time.	The values of adhesive and plasma-treated groups were significantly higher than the reference (untreated) group, whereas no statistical difference was found between the adhesive and plasma groups.
Zhou et al, 2014	PEEK reinforced with 7 wt% Nano-SiO_2_ (Jilin University Super Engineering Plastics Research)	PEEK + resin cements (RelyX Unicem, 3M Oral Care; or two-step SE Bond/ Clearfil AP-X, Kuraray)	Shear bond strength Cylinder dimensions: diameter: 3 mm; height: 4 mm Approach unclear*	Untreated condition	98% sulfuric acid (60 s) 9.5% hydrofluoric acid (60 s) Argon plasma treatment (25 min) Air abrasion with alumina particles (50-µm grain size, 15 s).	Storage: 24 h in distilled water at 37°C.	Regardless of surface treatment, the two-step system (SE Bond/Clearfil AP-X) promoted the highest bond strength. Sulfuric acid promoted the highest values regardless of the resin cement used, followed by argon plasma. Air abrasion, hydrofluoric acid and untreated groups showed lower or no bond between PEEK and resin cement.
Zhou et al, 2017	PEEK (Jilin University Super Engineering Plastics Research)	PEEK + adhesive (SE Bond, Kuraray) + resin cement (Clearfil AP-X, Kuraray)	Shear bond strength Cylinder dimensions: diameter: 3 mm; height: 4 mm Approach unclear*	Untreated condition	Air abrasion with alumina (50-µm grain size, 15 s); Argon gas plasma (25 min, 30 Pa, 13.56 MHz) Laser pretreatment, scanning speed of 1 mm/s, 1000 Hz, and pulse length of 800 nm, 20 mW, 100 fs of wavelength.	Storage: 24 h in distilled water at 37°C; one group was also incubated in distilled water (56 h at 37ºC) Or thermocycling for 5000 or 10,000 cycles at temperatures between 5ºC and 55ºC, 30-s dwell time.	Pretreatment of the PEEK surfaces with air abrasion, argon plasma, and laser improved the bond strength between PEEK and resin cement. Plasma treatment showed higher shear bond strengths, followed by air abrasion and laser. The bond strength of PEEK was decreased by the thermocycling conditioning methods (lowest values for 10,000 cycles of thermocycling).


**Fig 2:**
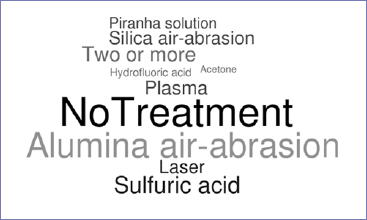
Word Cloud for surface treatments performed by the included studies. Larger and darker words represent the most frequently used treatments. Two or more: combination of different treatments; piranha solution: 98% sulfuric acid + 30% hydrogen peroxide.

The different surface treatments employed in the included studies are illustrated in Fig 2 and described in Table 3. Air abrasion with alumina using different parameters – eg, pressure (0.05 MPa, 0.2 MPa, 0.4 MPa, 2 Bar, 2.8 Bar), time (10 and 15 s), and grain size (45, 50, 110, 120 μm) – was the most common treatment for PEEK (19 studies), followed by sulfuric acid etching (11 studies) for different durations (5, 15, 30, 60, 90, 120 and 300 s) and at different concentrations (70%, 80%, 85%, 90%, and 98%), plasma (8 studies), laser (7 studies), tribochemical silica coating (7 studies using different grain sizes, 30 and 110 μm), piranha solution (5 studies), 9.5% hydrofluoric acid (2 studies), and acetone (1 study). The untreated condition (which was usually used as control) was reported in 23 studies. A combination of the mentioned treatments was also reported (9 studies) in evaluating physical/chemical approaches (tribochemical silica coating; alumina-particle air abrasion followed by piranha solution or plasma or laser; sulfuric acid etching + laser treatment). Despite the high heterogeneity of surface treatments explored, all studies were agreed on the importance of surface modification of PEEK to enhance its micromechanical bonding with resin-based materials. Sulfuric acid etching generally provided higher bond strengths (7 studies) when compared with other approaches, although one study^22^ reported no statistically significant difference between sulfuric acid etching and alumina particle air abrasion or with tribochemical silica coating.

Another factor which varied among the included in vitro studies (Table 3) was the use of different resin-based materials: veneering composite resin (11 studies) and resin cements (18 studies, 7 of which were self-etch). Thus, almost all studies considered the use of surface treatments in combination with adhesives. Among the studies which explored the use of adhesives, all reported a bond improvement compared with the no-treatment condition, corroborating that this step plays an important role for resin bond improvement to PEEK. Additionally, most studies reported that the use of a certain one-step adhesive (Visio.link, Bredent) containing methyl methacrylate (MMA), pentaerythritol triacrylate (PETIA), and dimethacrylates promoted bond enhancement when compared with adhesives with different compositions (8 studies). Thus, the composition of the adhesive on the obtained adhesion also seems to be influential.

Regarding the applied methodologies (Table 3), 22 studies used shear bond strength testing, while 7 studies adopted tensile test geometry. All specimens were cylindrical, with diameters ranging between 2.9 and 4 mm (just one study provided no clear description). Almost all studies (27 studies) mentioned at least 24 h of water storage, except for 2 studies which did not mention any water storage. Some studies evaluated the effect of aging protocols (4 studies with 14, 28, 30, 60, 90 or 150 days of water storage, and/or thermocycling 5 to 55°C for 5000, 10000, 12,000 or 37,000 cycles before bond strength tests in 6 studies) compared to baseline conditions. Almost all of these studies showed that aging protocols decreased the PEEK-resin bond strength, but 3 studies showed increased bond strength or no effect with these approaches.

Regarding failure analysis (Table 3), most of the studies (25) predominantly reported adhesive failures, except for a few studies which showed more mixed and/or cohesive failures for the resin-based material (2 studies). However, mainly cohesive/mixed failures were detected for specimens etched with sulfuric acid or its combination with alumina-particle air abrasion as pre-treatment (8 studies). Pre-test adhesive failures were only reported in 4 studies, which showed the occurrence of such failures during storage or when adjusting the specimens’ positioning for the bond strength test, especially in untreated control groups.

## Discussion

Promoting good adhesion to PEEK is a great challenge, since this material presents an inert behavior with low surface energy and resistance to surface modification,^19^ especially when PEEK is not filled with other substances, such as titanium oxide or silica. However, according to the collected data, a consensus seems to exist that surface modification of PEEK before bonding to resin-based materials is a requirement for achieving better bond strength, with sulfuric acid etching and air abrasion with alumina particles being the most effective options, mainly when combined with one certain adhesive (Visio.link, Bredent GmbH & Co KG) containing MMA, PETIA, and dimethacrylates.

In terms of surface treatment protocols, 98% sulfuric acid etching was the most effective to increase bonding to PEEK in the majority of the reports.^8,25,28,29,30,38,41^ Indeed, PEEK can be eroded by such treatment, and the resulting topographical changes provide microretentions for infiltration/filling with bonding agents (interlocking and micromechanical bond), thus increasing the bond strength.^41^ Furthermore, a previous study reported more cohesive failures after sulfuric acid etching, as well as the highest bond strengths.^14^ However, it is very important to note that sulfuric acid is hazardous and has a high corrosive risk.

In terms of acid concentration and etching duration when using sulfuric acid, previous studies showed that the most common concentration was 98% and 30- and 60-s etching duration was the most effective to increase the bond strength; however, both under-conditioning (shorter times or concentrations lower than 80% are ineffective to promote surface modification and resin tag penetration) and over-conditioning (longer etching times combined with the high corrosive effect of the acid leading to the deterioration of the material) could decrease PEEK’s adhesive ability.^7,29^ Thus, based on such hazardous potential and toxicity, sulfuric acid etching is not suitable for clinical applications, and its use in the laboratory environment demands caution. In addition, Rocha et al^22^ reported no difference between sulfuric acid and alumina air abrasion, which is safer for clinical use.

Among the surface treatments described by the included studies, the most common method employed to increase the bond strength to PEEK was alumina-particle air abrasion, which may promote a rougher, irregular surface, enabling mechanical interlocking between PEEK, the bonding agent, and the resin-based material.^2^ Although Tsuka et al^35^ reported that air abrasion with 50-μm alumina (10 s) induced resin bond strength similar to that of untreated PEEK, the reviewed studies generally corroborated that such treatment is one of the best options for bond promotion to PEEK,^2,6,8,9,16^ regardless of grain size, time, and pressure.

Tribochemical silica coating was also reported by some studies as another viable pretreatment for PEEK. This method not only makes surface topographical changes for micromechanical bonds, but also creates a silicon-oxide coating for chemical bonding to methacryloxypropyltrimethoxysilane coupling agent.^6^ However, almost all of the included studies showed comparable results between the tribochemical silica coating method and alumina-particle air abrasion alone,^2,6,8,22^ indicating no additional benefits of tribosilicatization over alumina particle abrasion for promoting adhesion to PEEK.

In the studies included here, laser and plasma treatments received less attention than air-abrasion protocols and sulfuric acid etching.^4,8,15,27,37^ Nevertheless, promising bond results were generally not found in comparison with alumina-particle air abrasion. In fact, some studies did not report any statistically significant difference between laser or plasma treatments and no surface treatments.^2,6,31^ Moreover, these methods are very expensive, demand additional infrastructure to perform, and are still uncommon in restorative clinical practice, corroborating the preference for other options.

The use of piranha solution (mixture of 98% sulfuric acid and 30% hydrogen peroxide) for etching was also reported.^12,16^ Piranha solution can increase surface roughness and improve the number of functional groups to interact with adhesives through the atomic oxygen released by hydrogen peroxide during the reaction with sulfuric acid, which reacts with benzene ring of PEEK.^12,32^ This treatment may also generate a topography of small pits on PEEK surfaces to be filled by bonding agents/resinous materials, increasing the bond strength.^28^ Another acid-etching treatment reported by the included studies was hydrofluoric acid; however, this approach was only evaluated by two studies.^38,41^ According to Yan et al,^38^ 9.5% hydrofluoric acid etching for 2 min was not able to promote significant surface micromorphological alterations for mechanical interlocking between PEEK and resin cement, generating low bond strengths, which is corroborated by the findings of Zhou et al.^41^

Adhesive agents play a highly relevant role in promoting adhesion to PEEK. The adhesive composition must also be taken into account when bonding PEEK to resin-based materials.^9^ For instance, there is evidence that Visio.link (Bredent) – which contains methylmethacrylate (MMA), dimethacrylates, and pentaerythritol triacrylate (PETIA) and is used with polymethyl methacrylate (PMMA) materials and high-performance polymers – may increase the bond strength between PEEK and resin-matrix to a greater extent than other adhesives with different compositions,^6,16,32^ after air abrasion and sulfuric acid etching. It seems that such components are able to increase the wettability of the modified PEEK surface and make it more reactive for bonding with resin-based material.^6,16^ Another study showed that there was no optimization of bond strength between PEEK and resin cement when using a silane bonding agent alone on surface-treated PEEK,^22^ thus corroborating the importance of using an additional adhesive after the surface treatment.

The included studies utilized various testing geometries to evaluate the bond strength between PEEK and resin-based materials. Macroshear was the most common test set-up, probably due to its simplicity and no need for sophisticated equipment.^5^ Furthermore, many of the studies reported using aging methods, including water storage and/or thermocycling,^2,10,23^ which generally showed that they generated a decrease in bond strength for PEEK. This may be explained by the detrimental effect of water on the bonded interfaces and resin materials,^13^ degrading the adhesive zone. It is also important to mention that pre-test failures were reported by some studies, confirming that the bonding ability of PEEK is poor, mainly when no surface treatment is performed.^12,19,22^ Finally, the failure pattern of samples subjected to bond testing must be highlighted during data interpretation, as it is widely known that cohesive failures represent unrealistic bond strengths, leading to a misinterpreted bond outcome (overestimation).^5^ In this sense, bond strengths obtained from cohesive failures must be carefully and critically evaluated and discussed.

Based on all of the aforementioned assumptions, it becomes clear that the combination of physical/mechanical and chemical conditioning methods is mandatory for improving resin bond strength to PEEK, as alumina-particle air abrasion followed by application of an adhesive containing MMA, PETIA and dimethacrylates (eg, Visio.link, Bredent) is one of the methods that produced the highest bond strengths. The former approach poses a much lower hazard than does sulfuric acid, which is the other most commonly reported effective surface treatment.^6,11^ However, it should be emphasized that studies evaluating sulfuric acid use in clinical scenarios are non-existent, and although in vitro studies are the only means of evaluating bond strength as an isolated factor, the absence of clinical studies evaluating its behavior under different stimuli and survival rates should be seen as a limitation of the current study. On the other hand, we emphasize that the present scoping review was effective to compile the whole available information regarding the use of surface treatments to increase resin bond strength to PEEK, and succeeded in confirming a promising protocol to do so.

## Conclusion

The findings of the present study allow the following conclusions:

- The combination of surface treatments and specific adhesives are essential to increase the bond strength between PEEK and resin-based materials.
- Sulfuric acid and alumina-particle air abrasion were the most effective surface treatments for promoting adhesion to PEEK.
- Alumina-particle air abrasion may be considered the preferential/optimal choice for PEEK surface treatment, since it promotes high bonding and is safer for clinical use than is sulfuric acid etching.
- A specific adhesive for PMMA and high-performance polymers (Visio.link, Bredent), containing MMA, PETIA, and dimethacrylates, promotes higher bond strength to PEEK.

## Clinical relevance:

Sulfuric acid etching and air abrasion with alumina particles were the most effective treatments to promote adhesion to PEEK, with air abrasion being safer for clinical use. The use of an adhesive containing MMA, PETIA, and dimethacrylates resulted in higher adhesion to surface treated PEEK.
